# Eating disorder risk assessment and sociocultural attitudes toward body image among Polish and Turkish professional female football players

**DOI:** 10.3389/fnut.2024.1456782

**Published:** 2024-09-18

**Authors:** Wiktoria Staśkiewicz-Bartecka, Samet Aktaş, Grzegorz Zydek, Marek Kardas, Martyna Kałuża, Małgorzata Magdalena Michalczyk

**Affiliations:** ^1^Department of Food Technology and Quality Evaluation, Department of Dietetics, Faculty of Public Health in Bytom, Medical University of Silesia in Katowice, Zabrze, Poland; ^2^Institute of Sport Sciences, Jerzy Kukuczka Academy of Physical Education in Katowice, Katowice, Poland; ^3^High School of Physical Education and Sports, Batman University, Batman, Türkiye; ^4^Department of Sport Nutrition, Jerzy Kukuczka Academy of Physical Education in Katowice, Katowice, Poland; ^5^Nutrition and Sports Performance Research Group, The Jerzy Kukuczka Academy of Physical Education in Katowice, Katowice, Poland

**Keywords:** eating disorders, female football player, body image, professional sport, sport nutrition

## Abstract

**Introduction:**

Football requires high physical fitness and often involves maintaining a specific physique, leading to harmful eating behaviors due to internal and external pressures. Cultural norms influence the prevalence and types of eating disorders (ED), with differences in eating habits, beauty standards, and socio-cultural pressures affecting ED development. This study assesses ED risk and analyzes socio-cultural attitudes toward body image among Polish and Turkish female football players.

**Methods:**

The study was conducted from April to June 2024 and included 103 professional female football players aged 18–35 from Polish (*n* = 49) and Turkish (*n* = 54) clubs. Data were collected using the CAWI method via Google Forms. Participants completed EAT-26 and SATAQ-3 questionnaires, assessing ED risk and socio-cultural attitudes toward body image.

**Results:**

40.8% of participants were at risk for ED. No differences in ED prevalence were found between groups of different nationalities. Polish footballers at risk of ED scored higher on the Internalization-Pressure and Information subscales and the overall SATAQ-3 score. A weak but significant positive correlation was found between SATAQ-3 and EAT-26 scores.

**Discussion:**

The study finds no nationality-based differences in ED prevalence among professional female football players, but ED is widespread, affecting about 40% of players. Nutritional status impacts ED risk, with higher risk among underweight and overweight players. Polish players are more influenced by socio-cultural standards of body image promoted by mass media. The study underscores the need for regular screening and psychological support.

## 1 Introduction

Football requires a high level of physical fitness, often necessitating the maintenance of a specific physique (1). Athletes face both internal and external pressures, including from teammates, coaches, and sports systems, to achieve and maintain a particular body shape for optimal performance and to meet esthetic ideals (2–5). These pressures can lead to overtraining and harmful eating behaviors, such as dietary restrictions and self-induced vomiting, particularly among women who face additional appearance-related pressures (2, 3, 6, 7). Social media further exacerbates these issues by promoting unrealistic beauty standards (8). Although there is evidence linking social media use to eating disorder (ED) prevalence among football players, there is a lack of research specifically on female players (9).

Eating disorders are behavioral disorders characterized by excessive weight control and obsessive thinking about food (8). According to American Psychiatric Association’s classification (DSM-V), these disorders are categorized into specific (e.g., anorexia nervosa, bulimia nervosa) and non-specific types, which include behaviors like emotional eating or food fanaticism (10). The International Statistical Classification of Diseases and Health Problems (ICD-11) classifies eating disorders as mental disorders linked to physiological and psychological factors, often accompanied by anxiety, depression, obsessive-compulsive disorder, and reduced quality of life (11). These disorders lead to harmful eating behaviors, such as starvation, frequent meal skipping, overeating followed by purging, and excessive exercise (12). Abnormal eating behaviors and overtraining in female athletes can lead to Relative Energy Deficiency in Sport (RED-S), a syndrome marked by low energy availability due to an imbalance between energy expenditure and intake (13, 14). RED-S, which evolved from the “female athlete triad,” is associated with injuries, reduced athletic performance, and serious health issues (13, 14).

Although there is extensive literature on ED in athletes, results are inconclusive. Some studies report higher levels of ED psychopathology in athletes compared to non-athletes, while others find no difference or the opposite effect (15). Differences in aspects such as body dissatisfaction, striving for thinness, and food restriction complicate the prioritization of research and prevention efforts without identifying specific at-risk sports and sociocultural factors (16–18).

ED are well-documented among athletes in esthetic sports, where they are judged on aspects like harmony of movement, physical attractiveness, and thinness (19, 20). However, recent studies show that ED also occur in non-esthetic sports, including individual sports like running, triathlon, cycling, and sports with weight categories (21–25). Therefore, it may be more appropriate to categorize sports as lean and non-lean (25).

Ball sports, where athletes’ weight and size do not directly affect performance, are classified as non-lean sports (25, 26). Despite this, studies show the presence of eating disorders (ED) among female footballers and volleyball players (9, 27). For example, Staśkiewicz et al. (9) found that many football players are at risk of developing ED, and Torres-McGehee et al. (28) reported that 25% of female football players experienced overeating. ED can occur in both lean esthetic and non-esthetic sports, including ball sports.

In the literature on the risk of ED among athletes, there are increasing inquiries about whether the risk of developing these disorders varies depending on the position of players on the field and their injury history. For instance, research by Torres-McGehee et al. has shown that athletes who have undergone periods of rehabilitation after injuries often exhibit higher levels of anxiety about their body and weight (28). Prolonged inactivity may lead to concerns about losing physical fitness, which in turn increases the risk of developing unhealthy eating patterns, such as excessive caloric restriction or intensive exercise to quickly regain fitness. On the other hand, research by Escobar-Molina et al. found that athletes participating in sports with a higher risk of injury are more susceptible to eating disorders due to the pressure to return to training and competition as quickly as possible (24). The rehabilitation process can lead to an obsession with weight and physical performance, particularly among athletes who fear losing their place on the team roster.

Additionally, the position of an athlete on the field can significantly influence the perceived risk of eating disorders. Studies on athletes playing different positions in football have shown that forward are often more exposed to pressure related to appearance and weight compared to defenders or goalkeepers (21). This group of players often strives to maintain a low body fat percentage, which is associated with the pressure to maintain a specific physique and physical fitness, especially in sports where speed and agility play a key role. Further research, such as that conducted by Sundgot-Borgen and Torstveit, indicated that female athletes in certain field positions, particularly those in more physically demanding roles, may be more susceptible to eating disorders (29). These positions require maintaining low body weight and high endurance, which can lead to unhealthy dietary practices to meet these demands.

Nationality and culture significantly impact the assessment of ED, influencing beauty norms, social expectations, access to resources, and psychological aspects (30). Cultural differences in beauty standards and ethnic diversity in diets and rituals can affect ED types and prevalence (31). Studies highlight differences in ED psychopathology between Western and non-Western societies, emphasizing the need for culturally sensitive assessment tools (32). The rise of ED in non-Western countries challenges the view that they are solely a Western issue, revealing complex cultural interactions (33). Western societies show higher levels of body dissatisfaction and general psychopathology than non-Western countries (34). Cultural pressures and media portrayals of ideal bodies further complicate the relationship between culture and ED development (35). Effective ED assessment requires considering cultural context to provide appropriate diagnosis and treatment.

Sociocultural theory provides a framework for exploring how environmental influences affect body image concerns. The Sociocultural Attitudes Toward Appearance Questionnaire (SATAQ-3) measures awareness and internalization of societal influences on appearance (36). Research consistently supports a strong relationship between sociocultural influences and body dissatisfaction (37).

The purpose of the study is to assess the risk of ED and analyze sociocultural attitudes toward body image among Polish and Turkish female football players. The study aims to identify differences in ED risk levels and attitudes toward beauty standards and social pressures between the two groups, taking into account the influence of nationality on these phenomena.

The study hypothesized that (1) female football players are at significant risk of eating disorders, furthermore (2) female football players from Poland and Turkey differ in their risk of eating disorders (3) there are differences in socio-cultural attitudes toward body image between the groups.

## 2 Materials and methods

### 2.1 The procedure of the survey

The survey was conducted from April to June 2024. Utilizing the CAWI (Computer-Assisted Web Interview) method, data was collected via a web form, an accepted approach in psychological research. Google Forms was chosen for its user-friendliness, accessibility, and customizability. Athletes were given a QR code before the start of the training session that directed them to the survey. Researchers explained the process beforehand to ensure proper understanding and minimize errors. The female football players completed the survey as soon as they received the QR code, and no time limit was set for completing the questionnaire.

The study used purposive sampling. With this method, the sample was selected to represent characteristics and specific experiences related to the topic of the study. Determining precise selection criteria, such as gender, sports discipline, and nationality, was crucial to achieving the study’s objectives.

Participants in the study were informed about the purpose of the study and its anonymity and were asked to accept the rules of data sharing. Information about informed and voluntary participation in the study was at the beginning of the questionnaire. The World Medical Association’s Declaration of Helsinki guided the conduct of this study. The study was approved by the Bioethics Committee of the Silesian Medical University in Katowice (BNW/NWN/0043–3/641/35/23, date of approval: 22/09/2023) in light of the Law of December 5, 1996 on the Profession of Physician and Dentist (Journal of Laws 2016, item 727).

### 2.2 Study participants

The study involved 103 female football players aged 18–35 from 4 sports clubs, 2 clubs were located in Poland (*n* = 49) and 2 in Turkey (*n* = 54). Based on the location of the clubs, the players were divided into two groups: Polish female football players (PL) and Turkish female football players (TR). The Polish players competed in the Ekstraliga (1st level of play) and I Liga (2nd level of play), while the Turkish players participated in the Women’s 2nd league (3rd level of play) and Women’s 3rd league (4th level of play). All clubs participating in the study, according to the statutes of the Polish Football Association (PZPN) and the Turkish Football Federation (TFF), participate in professional football competitions (38, 39). The response rate for the PL group was 90.74%, and for the TR group, it was 96.42%.

The study was conducted in the final phase of the starting spring round of the 2023/2024 league season, all players were tested during the same period of the training macrocycle to obtain comparable results.

The inclusion criteria for the study were as follows: (1) female gender (2) club consent for study participation, (3) voluntary participation, (4) age 18 years or older, (5) active player status at the time of the study, (6) no injuries leading to a training break of at least 7 days within the last 2 months, (7) proficiency in Polish or Turkish. The exclusion criterion was any incorrectly or incompletely completed questionnaire.

### 2.3 Research tools

A survey questionnaire was used to conduct the study, which consisted of a metric section (respondent’s data: age, height, weight, chronic diseases including mental disorders such as depression, eating disorders, neurosis, etc., medications taken, education, position held, sports experience, number of workouts per week, sources of nutritional knowledge, food exclusions, information on social media use) and questionnaires- Socio-Cultural Attitudes Questionnaire (SATAQ 3) Eating Attitudes Test (EAT-26).

#### 2.3.1 Body mass index

The nutritional status of the subjects was evaluated through the calculation of the body mass index (BMI). BMI was derived using the standard formula:


$$BMI=\frac{\mathrm{body}\;\mathrm{weight}\;\left(\mathrm{kg}\right)}{\mathrm{height}\;{\left(m\right)}^{2}}$$

The results were interpreted according to the World Health Organization (WHO) (40).

#### 2.3.2 EAT-26

The study used a screening tool to assess ED risk, the American Dietary Attitudes Test developed by Garner et al. 448. This questionnaire is a widely recognized standardized instrument used to identify symptoms indicative of ED risk. It is designed to screen individuals both with a clinical diagnosis and those at risk for AN, BN, or obesity. EAT-26 is one of the most extensively used diagnostic tools in global studies on the prevalence of eating disorders. The author of the Polish standardization of the tool is K. Wlodarczyk-Bisaga (41), while the Turkish standardization was developed by F. Elif Ergüney-Okumuş and H. Özlem Sertel-Berk (42). The test is not a diagnostic tool, but some authors have suggested that the EAT-26 can identify cases at risk for ED on the clinical spectrum (43).

The interpretation of the EAT-26 questionnaire consists of three “referral criteria” that determine whether the respondent should report for further evaluation of ED risk:

1. The final score on the EAT-26 questionnaire is calculated by summing the scores from 26 questions that evaluate attitudes toward nutrition. Questions 1 through 25 are scored as follows: Always = 3 points; Usually = 2 points; Often = 1 point; Other answers = 0 points. Question 26 is scored inversely: “Never” = 3 points, and so forth. The total score ranges from 0 to 78. A score of 20 or higher indicates a potential risk for an eating disorder, warranting consultation with a specialist for further evaluation.
2. Questions regarding behavioral patterns may indicate the presence of symptoms associated with ED or recent significant weight loss. These questions target compensatory behaviors such as the use of laxatives, self-induced vomiting, binge eating, excessive physical activity, and rapid and significant weight loss over a short period. An affirmative response to any of these questions might suggest abnormalities and necessitate further diagnostic evaluation for ED.
3. The survey contains specific questions about respondents’ height, body mass, and gender, which are essential for calculating the BMI. BMI can indicate potential risks of ED, particularly if the body mass is significantly low relative to age standards. By evaluating BMI alongside height, body mass, and gender data, it becomes possible to identify individuals at risk and highlight the need for further analysis. Interpretation of women’s BMI compared to norms for age (44).

The overall EAT-26 scale in the Polish version showed very good omega McDonald’s internal consistency at 0.811. For individual subscales, it was 0.790 for part A, 0.777 for part B. In contrast, the Turkish version of the EAT-26 scale showed satisfactory omega McDonald’s internal consistency of 0.727. For individual subscales, it was 0.742 for part A, 0.704 for part B.

#### 2.3.3 SATAQ-3

The Socio-Cultural Attitudes Toward Appearance Scale 3 (SATAQ 3) is a widely used method of measuring the influence of socio-cultural norms promoted by mass media on attitudes and behaviors regarding the body and physical appearance. The scale was developed by Heinberg and Thompson (45). The SATAQ 3 questionnaire and its earlier versions are useful methods for assessing the strength of pressure and internalization of socio-cultural standards of body image and physical appearance. Correlational studies have shown significant associations between the SATAQ 3 and measures of body image and ED indicators, such as body dissatisfaction and striving to be excessively thin (46). The questionnaire consists of 30 items to which the respondent should respond using a five-point Likert scale, where 1 means strongly disagree and 5 means strongly agree. The scale has four homogeneous factors: Internalization-General, Internalization- Athlete, Pressures and Information. Cronbach’s *α* coefficient calculated for the entire tool was 0.96 (45). The original internal structure of the SATAQ-3 has not been replicated in the different versions and adaptations (47).

The study used the Polish version of the questionnaire developed by Izydorczyk B. Lizińczyk S. (48) and the Turkish version developed by Swami V. et al. (49).

##### 2.3.3.1 Polish version

The results of factor analysis and unsatisfactorily low values of factor loadings for the identified factors in items #3 and #9 resulted in the exclusion of these items from the Polish version of the SATAQ-3 questionnaire, which contains 28 questions (46). Four factors obtained in the Polish version of the SATAQ-3 questionnaire were named differently than in the English version of the tool. Internalization-Pressure scale (12 items), Internalization-Information Seeking scale (6 items), Internalization-Athlete scale (4 items), Information scale (6 items). Cronbach’s *α* coefficients were satisfactory for the individual subscales of the tool in the surveyed population of Polish men and women. They ranged from 0.76 to 0.92. The Polish adaptation and normalization of the SATAQ-3 have similar reliability and statistical properties to the original version of the tool developed by Thompson et al. (42, 46).

The overall SATAQ scale in the Polish version showed very good internal consistency of McDonald’s omega at 0.927. For the “Internalization-Pression” scale it was 0.956 for the “Internalization-Athlete” scale it was 0.849 for the “Internalization-Information Seeking” scale it was 0.817 for the “Information scale” it was 0.831.

##### 2.3.3.2 Turkish version

The results of the analysis showed that item #20 appeared to cross-load two factors, this resulted in the exclusion of this item from the Turkish version of the SATAQ-3 questionnaire, which contains 29 questions (47). The Turkish version of the SATAQ-3 questionnaire has four factors: Information (9 items), Pressure (7 items), Internalization-General (9 items), and Internalization-Athlete (4 items) and internal consistency of the scale in was 0.93, while it ranged between 0.70 and 0.91 for the subscales. The Turkish adaptation and normalization of the SATAQ-3 have similar reliability and statistical properties to the original version of the tool developed by Thompson et al. (45, 48, 49).

The overall SATAQ scale in the Turkish version showed very good internal consistency of McDonald’s omega at 0.923. For the “Internalization- General” scale it was 0.828 for the “Pressure” scale it was 0.888 for the “Internalization-Athlete” scale it was 0.734 for the “Information” scale it was 0.846.

### 2.4 Statistical analysis

Statistical analyses were performed using Statistica v.13.3 (Stat Soft Poland) and the R package v. 4.0.0 (2020) under the GNU GPL (The R Foundation for Statistical Computing). To present quantitative data, mean values and standard deviations (X ± S) were calculated; for qualitative data, percentage notation was used.

The statistical analysis used the Chi^2^ and Fisher’s exact test to compare groups. The Chi^2^ test was used to evaluate differences between groups for categorical variables such as education level, sources of nutrition knowledge, and frequency of additional training outside the sports club. Fisher’s exact test was used in situations where the sample sizes in each category were too small for the Chi-square test to be meaningful. The significance of differences between female football players of different origins was assessed using Student’s t-test for two parametric groups, analysis of variance (ANOVA) for three or more parametric groups, Mann–Whitney U test for two non-parametric groups, and Kruskal-Wallis test for three or more non-parametric groups. Pairwise comparisons of body weight measurements were made using the Durbin-Conover test.

To examine the relationship between EAT-26 and SATAQ-3 scores, the Spearman Correlation Coefficient was used. This coefficient measures the strength and direction of the relationship between two ordinal variables.

A linear regression analysis was conducted to assess the relationship between various dimensions of sociocultural internalization and the EAT-26 score, which measures the risk of ED. The results of the analysis are presented in the form of regression coefficients (Estimates), along with standard deviations, t-statistics, and statistical significance levels.

A value of *p* < 0.05 was used as a criterion for statistical significance.

## 3 Results

### 3.1 Characteristic study group

One hundred and three female football players participated in the study after considering the inclusion and exclusion criteria. Based on their club origin, the players were divided into two groups: the first group (*n* = 49) consisted of female players from Polish clubs (PL), while the second group (*n* = 54) consisted of female players from clubs located in Turkey (TR). One female football player had a chronic illness (allergy) and was taking medication (Flixonase). The educational background of the participants varied between the two groups. Female athletes from Poland had primary (*N* = 11), secondary (*n* = 29), and higher education (*n* = 9), while Turkish women had only secondary (*n* = 9) and higher education (*n* = 45; *p* < 0.001). There were differences in the sources of nutritional knowledge among the female athletes. The Polish women drew their knowledge mainly from the internet (*n* = 25) and from a nutritionist (*n* = 11), while the Turkish women obtained theirs from a coach (*n* = 22) and the internet (*n* = 17; *p* < 0.001). No significant differences were observed in performing additional training units outside the sports club (*p* = 0.559), with the majority of female athletes declaring that they train 1–2 times per week (*n* = 44) or 3–4 times per week (*n* = 35). According to the interpretation of the BMI index, one female athlete from Poland was overweight, while five female athletes from Turkey were underweight (*p* = 0.056).

The players were also asked about their lowest and highest body weight in adulthood, as well as what they thought was the ideal body There was a statistically significant difference between PL and TR female football players in the question about the highest body weight (*p* = 0.017). In addition, there was a statistically significant difference between the highest (*p* < 0.001), lowest (*p* < 0.001), and ideal (*p* = 0.002) body weight and current body weight for the entire study group (Table 1).

**Table 1:** Characteristics of the study group, including the current, lowest, highest, and considered ideal body mass of female football players (*n* = 103).

	Total (*n* = 103)	PL (*n* = 59)	TR (*n* = 54)	*p*-value
Age [years]	21.80 ± 3.47	22.20 ± 4.15	21.40 ± 2.70	0.295
Height [cm]	166.00 ± 6.16	168.00 ± 5.81	155.00 ± 6.09	0.006*
Body mass [kg]	58.10 ± 6.31	60.50 ± 5.76	55.90 ± 6.03	<0.001*
BMI [kg/m^2^]	21.00 ± 1.61	21.40 ± 1.28	20.60 ± 1.79	0.010*
Body mass; CURRENT	58.10 ± 6.31	60.50 ± 5.76	55.90 ± 6.03	0.257
Body mass; LOWEST	53.80 ± 7.29	56.80 ± 6.26	51.20 ± 7.17	0.393
Body mass; HIGHEST	61.70 ± 7.48	64.1 ± 7.38	59.6 ± 6.96	0.017*
Body mass; IDEAL	57.70 ± 5.45	59.30 ± 5.46	56.20 ± 5.05	0.006*
CURRENT – HIGHEST *p* < 0.001* CURRENT – LOWEST *p* < 0.001* CURRENT – IDEAL *p* = 0.002*				

All data are presented in the table as mean and standard deviation (X ± SD); BMI, Body Mass Index; PL, Polish female football player; TR, Turkish female football player; *p < 0.05.

### 3.2 Risk of ED

Based on the results of the EAT-26, Part A questionnaire, it was estimated that 11.7% of the respondents (both Polish and Turkish women) were at risk for ED and should seek further diagnosis from a specialist. There were no significant differences between the groups in the total EAT-26, Part A test score, which may indicate the risk of developing eating disorders (*p* = 0.242). Additionally, there were no statistically significant differences between the groups in terms of nutritional status as interpreted by BMI value according to WHO recommendations (*p* = 0.787), education level (*p* = 0.208), and the amount of additional training outside the sports club (*p* = 0.123), about the total score of the EAT-26, Part A test.

According to the accepted results in the behavioral questions from the EAT-26 test, Part B, it was estimated that 28.6% of Polish and 33.3% of Turkish female football players met the criterion that may indicate a risk of developing ED. There was no significant effect of nationality on EAT-26 test scores for behavioral questions (*p* = 0.602). Additionally, no statistically significant differences were found between groups in terms of nutritional status as interpreted through BMI values according to WHO recommendations (*p* = 0.291), education level (*p* = 0.637), and the amount of additional training outside the sports club (*p* = 0.192) concerning the total EAT-26 Part B test score.

According to the accepted norms in Part C of the EAT-26 test (low body weight compared to age norms), it was estimated that 2% of Polish and 16.7% of Turkish female football players met a criterion that may indicate a risk of developing an ED. There was a significant effect of nationality on the results of Part C of the EAT-26 test (*p* = 0.012). Additionally, no statistically significant differences were found between the groups in terms of education level (*p* = 0.162) and the amount of additional training outside the sports club (*p* = 0.345) to the EAT-26 Part C test score.

Based on the overall results and interpretation of the EAT-26 questionnaire, it was found that 40.8% of the female respondents (both Polish and Turkish) met at least one of the three criteria that may indicate the likely existence or susceptibility to ED. These individuals should see a specialist for further diagnosis. There was no significant effect of the level of origin on the overall EAT-26 score (*p* = 0.231). Statistically significant differences were found between nutritional status interpreted through BMI values according to WHO recommendations and the total EAT-26 score (*p* = 0.004). Overweight and underweight athletes were more likely to have an increased risk of ED. There was no relationship between education level and the total EAT-26 test score (*p* = 0.416). However, there was a correlation between the frequency of extra training outside of the football club and the total EAT-26 test score (*p* = 0.025). The conducted study did not show a statistically significant relationship between the players’ position on the field and the risk of ED, as assessed by the EAT-26 scale. The result of the statistical analysis (*p* = 0.328) suggests that the position played by the athlete does not significantly influence the risk of developing eating disorders (Table 2).

**Table 2:** Summary of ED risk estimation (EAT-26; *n* = 103).

EAT-26	Total (103)		PL (*n* = 49)		TR (*n* = 54)		*p*-value
Elevated risk	No risk	Elevated risk	No risk	Elevated risk	No risk	Elevated risk	
Part A n (%)	91 (88.3)	12 (11.7)	43 (87.8)	6 (12.2)	48 (88.9)	6 (11.1)	*0.858*
Part B n (%)	71 (68.9)	32 (31.1)	35 (71.4)	14 (28.6)	36 (66.7)	18 (33.3)	0.602
Part C n (%)	93 (90.3)	10 (9.7)	48 (98.0)	1 (2.0)	45 (83.3)	9 (16.7)	0.012*
Entire n (%)	61 (59.2)	42 (40.8)	32 (65.3)	17 (34.7)	29 (53.7)	25 (46.3)	0.231

PL, Polish female football player; TR, Turkish female football player; *p < 0.05.

### 3.3 Influence of sociocultural attitudes toward appearance on ED risks

Analyzing the results obtained using the SATAQ-3, significant statistical relationships were found in the group of Polish athletes. Football players at risk of developing ED scored higher in the sections Internalization-Pressure (26.5 ± 12.2 vs. 18.3 ± 7.04, *p* = 0.010), Information (13.6 ± 5.05 vs. 10.5 ± 3.54, *p* = 0.041), and the total scale (69.8 ± 17.8 vs. 56.8 ± 15.3, *p* = 0.011). In the group of Turkish athletes, no statistically significant relationships were found between the scores in individual sections and the risk of developing ED. Additionally, significant statistical differences were found between the groups of Polish and Turkish athletes in the overall number of points obtained (61.3 ± 17.2 for the PL group vs. 73.1 ± 19.6 for the TR group, *p* = 0.002). Detailed information is presented in Table 3.

**Table 3:** Comparison of SATAQ-3 scores between Polish and Turkish female football players based on risk of developing ED (EAT-26).

	PL					TR			
Scale	Total (*n* = 49)	ER (*n* = 17)	NR (*n* = 32)	*p*-value	Scale	Total (*n* = 54)	ER (*n* = 25)	NR (*n* = 29)	*p*-value
I-P 12–60 point	21.2 ± 9.86	26.5 ± 12.2	18.3 ± 7.04	0.010*	P 7–35 points	15.7 ± 6.16	15.7 ± 5.59	15.8 ± 6.87	0.773
I-I S 6–30 points	17.7 ± 5.94	17.7 ± 4.01	17.7 ± 6.81	0.674	I-G 9–45 points	22.5 ± 5.61	22.6 ± 5.55	22.6 ± 5.76	0.986
I-A 4–20 points	10.9 ± 4.25	11.9 ± 3.94	10.3 ± 4.35	0.184	I-A 4–20 points	11.8 ± 3.80	11.6 ± 3.88	12.0 ± 3.77	0.564
I 6–30 points	11.6 ± 4.35	13.6 ± 5.05	10.5 ± 3.54	0.041*	I 9–45 points	23.1 ± 7.12	23.0 ± 7.67	23.2 ± 6.74	0.715
Total 28–140 points	61.3 ± 17.2	69.8 ± 17.8	56.8 ± 15.3	0.011*	Total 29–145 points	73.1 ± 19.6	72,8 ± 21.1	73.4 ± 18.5	0.609
*p*-value	0.002*								

All data are presented in the table as mean and standard deviation (X ± SD); PL, Polish female football player; TR, Turkish female football player; *p < 0.05; ER, Elevated Risk; NR, No Risk; *p < 0.05; I-P, Internalization-Pressure; I-I S, Internalization-Information Seeking; I-A, Internalization-Athlete; I, Information; P, Pressure; I-G, Internalization-General.

### 3.4 Relationship between SATAQ-3 score and EAT-26

**Figure 1:**
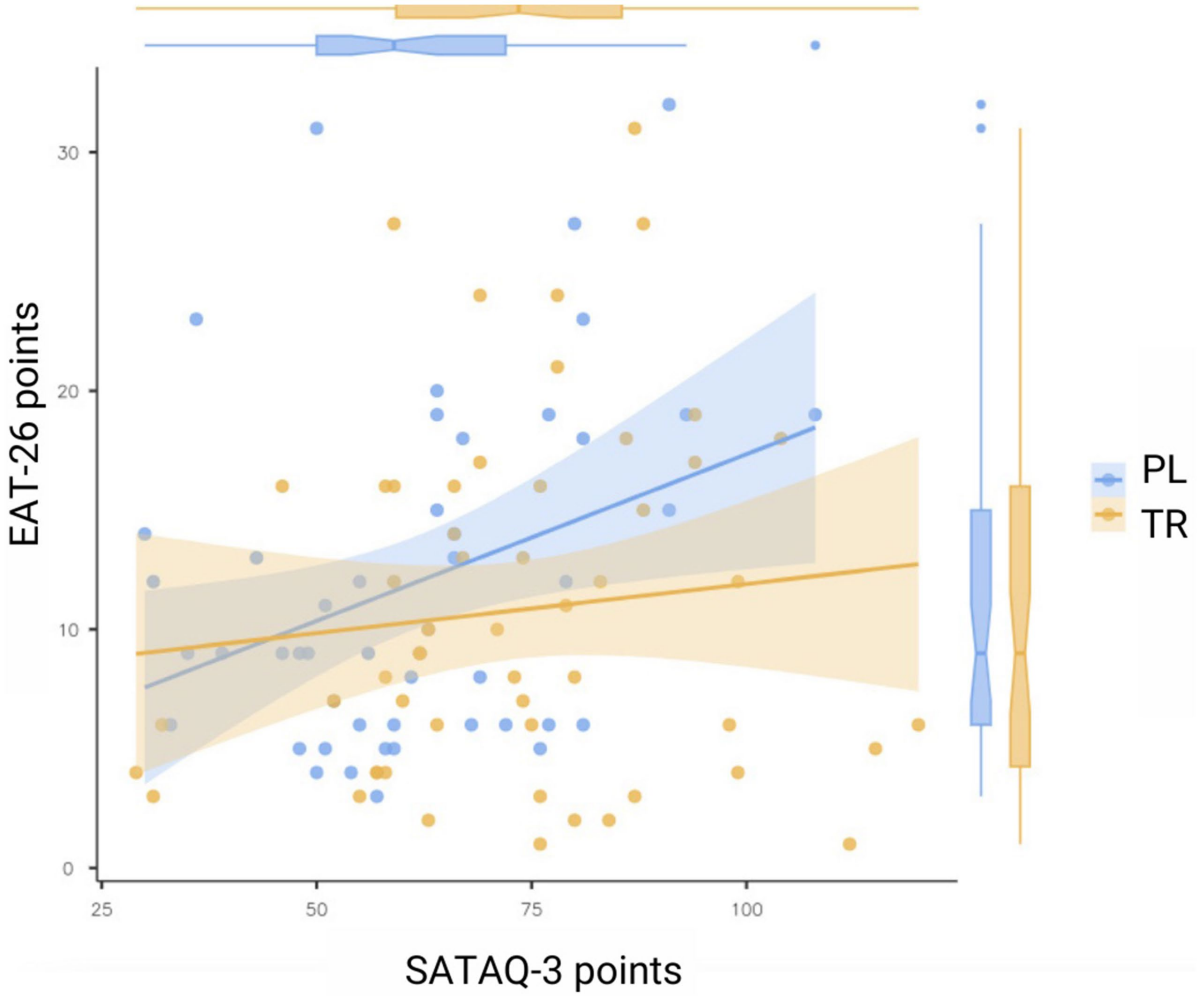
Statistical analysis of the relationship of scores in the SATAQ-3 and EAT-26 tests (*n* = 103).

The analysis revealed a statistically significant positive correlation between the scores of SATAQ-3 and EAT-26 (*p* = 0.036, *ρ* = 0.207). This indicates a weak but significant association, suggesting that higher scores on one test are related to higher scores on the other (Figure 1).

#### 3.4.1 Linear regression analysis of EAT-25 AND SATAQ-3 in PL group

The Table 4 presents the results of a linear regression analysis aimed at examining the relationship between various dimensions of sociocultural internalization (Internalization-Pressure, Internalization-Information Seeking, Internalization-Athlete, Information) and the EAT-26 score, which measures the risk of eating disorders. A significant effect on the EAT-26 score was observed for two variables: Internalization-Pressure (*p* < 0.001) and Information (*p* = 0.029). Internalization-Pressure had a positive impact on the score, indicating that greater internalization of societal pressure is associated with an increased risk of eating disorders. On the other hand, the Information variable had a negative effect, suggesting that access to information may reduce the risk of ED. The other variables, such as Internalization-Information Seeking and Internalization-Athlete, did not have a significant impact on the EAT-26 score (*p* > 0.05). The model’s fit measure (R^2^ = 0.33) indicates that approximately 33% of the variability in EAT-26 scores can be explained by these predictors.

**Table 4:** Linear regression analysis of sociocultural predictors and EAT-26 score in PL group (*n* = 49).

Model coefficients – EAT-26 score; *R* = 0.574; *R*^2^ = 0.330				
Predictor	Estimate	SE	t	*p*-value
Intercept	8.3347	3.673	2.269	0.028
Internalization-Pressure	0.5131	0.134	3.823	< 0.001
Internalization-Information Seeking	−0.0324	0.153	−0.212	0.833
Internalization-Athlete	0.0274	0.256	0.107	0.915
Information	−0.6030	0.267	−2.257	0.029

#### 3.4.2 Linear regression analysis of EAT-25 AND SATAQ-3 in TR group

The Table 5 presents the results of a linear regression analysis aimed at investigating the relationship between several sociocultural dimensions (Pressure, Internalization-General, Internalization-Athlete, Information) and the EAT-26 score, which measures the risk of eating disorders. The model shows that Internalization-General has a significant positive impact on the EAT-26 score (*p* = 0.049), indicating that higher internalization of general sociocultural standards is associated with an increased risk of eating disorders. The remaining variables were not statistically significant, indicating they do not have a notable impact on the EAT-26 score.

**Table 5:** Linear regression analysis of sociocultural predictors and EAT-26 score in TR group (*n* = 54).

Model coefficients – EAT-26 score; *R* = 0.306; *R*^2^ = 0.0938				
Predictor	Estimate	SE	t	*p*-value
Intercept	2.8540	4.461	0.640	0.525
Pressure	−0.5210	0.285	−1.828	0.074
Internalization-General	0.6846	0.339	2.022	0.049
Internalization-Athlete	−0.0876	0.364	−0.240	0.811
Information	0.0554	0.212	0.262	0.795

The model’s fit (R^2^ = 0.0938) suggests that approximately 9.38% of the variance in the EAT-26 score can be explained by the predictors included in the model, which indicates a relatively weak fit.

## 4 Discussion

Following Papathomas and Lavallee’s (50) call for greater methodological pluralism and focus on athletes from sports with traditionally low ED incidence, this study found that female football players are at significant risk for ED, challenging traditional classifications of high- and low-risk sports. The study aimed to assess ED risk and analyze socio-cultural attitudes toward body image among Polish and Turkish professional female football players, identifying differences in ED risk levels and attitudes toward beauty standards and social pressure, with a focus on the influence of nationality.

The high risk of ED among this group of athletes, as indicated by the EAT-26 results, is alarming, with an estimated 40.8% at risk. Although football is not typically associated with a high risk of ED, our findings suggest that Polish and Turkish female football players may be vulnerable. Assessing ED in this group is crucial due to the serious risks to both physical and mental health. ED can lead to severe nutritional deficiencies, negatively affecting physical performance and increasing injury risk. Long-term ED can shorten athletes’ careers and are often accompanied by psychological issues, potentially leading to depression, anxiety, and even suicidal thoughts.

The prevalence of ED among women can vary significantly by country, influenced by cultural, social, and economic factors, as well as the specific sports environments (51). Cultural beliefs and attitudes are key contributors to ED development, with prevalence differing across racial, ethnic, and national groups and evolving (52). Although ED are more widespread in various cultural groups than previously recognized, our study found no differences between the Polish and Turkish groups.

In the study by Abbot et al. (53), the prevalence of ED among football players was assessed using EAT-26, and it was shown that approximately 11% of the athletes, according to the test interpretation, are at risk for developing ED. In the study conducted by Izquierdo et al. (54), similar ED risk rates were reported, indicating that about 11% of men and women playing football were at risk or suffering from ED. However, in the only other study that assessed the prevalence of ED risk among elite female football players, the results were less alarming. In that study, none of the 36 professional athletes scored higher than the clinical cut-off point of 20 in EAT-26 (29). Our results indicate that the problem is significantly more widespread. Similar conclusions regarding the greater prevalence of these disorders were demonstrated in the study by Staśkiewicz-Bartecka and Kardas (9), where it was shown that 16.7% of male football players are at risk for ED.

In the present study, approximately 40.8% of female football players were identified as being at risk of developing ED, a figure significantly higher than those reported in previous studies such as Abbott et al., where the prevalence was approximately 11% (53). Several factors could explain this discrepancy. Firstly, the training status and season moment at which the study was conducted might have influenced the findings. Our study was carried out in the final phase of the spring round, a period that likely involves peak physical demands and psychological stress as players prepare for or compete in crucial matches. The pressures related to performance, maintaining fitness levels, and adhering to body image ideals during this phase could heighten the risk of disordered eating behaviors (29).

Furthermore, the higher percentages found in our study could also be attributed to cultural and environmental factors that differ between studies. For instance, the sociocultural influences on body image, particularly in Polish athletes, who scored higher on the SATAQ-3 subscales related to media pressure, may contribute to a greater internalization of appearance ideals, thereby increasing ED risk. The differences in training intensity, nutritional guidance, and psychological support available in the clubs studied compared to other research settings could also play a role. Future research should consider controlling for these variables to better understand their impact on ED prevalence among athletes.

Overall, the data suggest that the prevalence of ED is related to socio-cultural attitudes toward appearance. In our study, it was shown that individuals at risk for ED exhibited higher levels of socio-cultural attitudes toward appearance. The analysis showed that in the group of Polish athletes, significant statistical relationships were found between scores in the Internalization-Pressure (*p* = 0.010), Information (*p* = 0.041) sections, and the overall SATAQ-3 scale (*p* = 0.011). In the group of Turkish athletes, no such relationships were found. Furthermore, significant statistical differences between the two groups of athletes in the total number of points obtained in the SATAQ-3 scale (*p* = 0.002) indicate the diversity of cultural influences. The analysis revealed a statistically significant positive correlation between the EAT-26 and SATAQ-3 test scores (*p* = 0.036, *ρ* = 0.207). This means that there is a weak but significant relationship between the scores of these two tests, suggesting that higher scores in one test are associated with higher scores in the other. Although the correlation is significant, it is important to note that the correlation coefficient (ρ = 0.207) indicates a weak strength of this relationship.

These results support and extend the existing literature on the relationship between socio-cultural attitudes toward appearance and ED. In the study by Calogero et al. (55), higher SATAQ-3 scores were shown for patients with BN. Patients with BN scored higher than all other groups in the Internalization-Athlete section and higher in the General Internalization section than the AN groups. The means for the ED sample were higher than the means reported for non-ED individuals, except for the Information subscale. Correlations between the SATAQ-3 subscales and the ED assessment scale (EDI) indicated a positive association between the measures. The utility of the SATAQ-3 measure should be considered in any future research aimed at determining predictive factors for the occurrence of ED problems. The SATAQ-3 is reliable and valid in samples of individuals with ED and is an appropriate measure for use in this population (52). The study by Yessenia Lazo Montoya et al. (56) confirms our results. An association was observed between SATAQ-3 scores, both overall and subscale scores, and the risk of developing ED (EAT-26).

Over the past two decades, studies have documented an increase in the public’s desire for slim female figures. Greater media exposure is linked to more pronounced ED symptoms. The internalization of the thin ideal has been identified as a causal risk factor for ED and a significant predictor of success in prevention efforts (57).

The study posed three key hypotheses: (1) female football players are significantly at risk of eating disorders (ED), furthermore (2) female football players from Poland and Turkey differ in their risk of ED, and (3) there are differences in socio-cultural attitudes toward body image between the groups.

The results partially confirmed these hypotheses. About 40% of participants were at risk of ED, confirming the first hypothesis. However, no significant differences in ED risk were found between Polish and Turkish players, contradicting the second hypothesis. The third hypothesis was confirmed, as Polish players were more influenced by socio-cultural factors promoted by the media, while Turkish players showed no significant differences in this regard. This highlights the need for culturally tailored interventions and the importance of regular screening and psychological support for all female football players.

### 4.1 Strengths and limitations

The strengths of this study include a high participant response rate (90.74% for the Polish group and 96.42% for the Turkish group), which enhances the representativeness of the findings and reduces the risk of selection bias. Additionally, the study was conducted in the final phase of the spring round of the 2023/2024 league season, allowing for comparable results in the context of the athletes’ training phase. The study provides new information on cultural differences and their impact on the development of ED among professional female football players from different countries, which constitutes a valuable contribution to the existing scientific literature. This comparison is crucial for understanding how varying cultural contexts impact the mental and physical health of athletes, which is significant for global prevention and treatment strategies.

However, the study also has some limitations. Firstly, the use of a cross-sectional design prevents establishing causal relationships between variables. Future research should include a longitudinal component that would allow for tracking changes over time and more reliably determining the direction of influence. Additionally, the study sample consisted exclusively of professional female football players, which may limit the generalizability of the results to other groups of athletes or the general population. Another limitation is the possibility of self-report errors, which may affect the accuracy of the collected data, especially in the context of assessing body weight and height. Although well-validated research tools were used, cultural differences may affect the way questions in the questionnaires are interpreted and answered. One of the significant limitations of our study is the lack of detailed data on the participants’ body composition, such as body fat percentage, muscle mass, or water distribution, which could provide more precise information on health status and potential risks related to eating disorders. The BMI, although commonly used, does not account for differences in body composition, which may lead to an inaccurate assessment of nutritional status in individuals with higher muscle mass, typical of athletes. Moreover, our study lacks detailed information on nutritional parameters, such as dietary habits, intake of macro- and micronutrients, and access to appropriate dietary support. These data could provide valuable insights into the relationships between nutritional status and the risk of ED. Including this information in future studies could contribute to a more comprehensive understanding of the factors influencing the risk of developing ED in athletes.

## 5 Conclusion

The results of this study showed that professional female football players, regardless of nationality, are significantly at risk of ED, with approximately 40% of the players being at risk. It was demonstrated that nutritional status, interpreted through the BMI index, affects the risk of ED. Female football players with underweight and overweight had a higher risk of ED. Furthermore, differences were found in the influence of socio-cultural attitudes toward body image among players of different nationalities. Polish female football players are characterized by the higher influence of socio-cultural standards of body image and appearance promoted in the mass media. Polish players at risk of developing eating disorders scored higher in the subscales of Internalization-Pressure and Information, as well as in the overall SATAQ-3 test score. This relationship was not observed among Turkish players. The study found a weak but significant positive correlation between SATAQ-3 and EAT-26 scores, indicating that higher socio-cultural pressure and internalization are associated with a higher risk of developing ED. These results suggest the significant role of regular screenings and mental health support for professional female football players.

## Data Availability

The raw data supporting the conclusions of this article will be made available by the authors, without undue reservation.
